# Classifying lower grade glioma cases according to whole genome gene expression

**DOI:** 10.18632/oncotarget.12188

**Published:** 2016-09-22

**Authors:** Baoshi Chen, Tingyu Liang, Pei Yang, Haoyuan Wang, Yanwei Liu, Fan Yang, Gan You

**Affiliations:** ^1^ Department of Neurosurgery, Beijing Tiantan Hospital, Capital Medical University, Beijing, China; ^2^ Beijing Neurosurgical Institute, Capital Medical University, Beijing, China; ^3^ Chinese Glioma Cooperative Group (CGCG), China; ^4^ Department of Neurosurgery, Guangdong Zhujiang Hospital, Southern Medical University, Guangzhou, China

**Keywords:** lower grade glioma, risk score, gene signature, prognosis

## Abstract

**OBJECTIVE:**

To identify a gene-based signature as a novel prognostic model in lower grade gliomas.

**RESULTS:**

A gene signature developed from HOXA7, SLC2A4RG and MN1 could segregate patients into low and high risk score groups with different overall survival (OS), and was validated in TCGA RNA-seq and GSE16011 mRNA array datasets. Receiver operating characteristic (ROC) was performed to show that the three-gene signature was more sensitive and specific than histology, grade, age, IDH1 mutation and 1p/19q co-deletion. Gene Set Enrichment Analysis (GSEA) and GO analysis showed high-risk samples were associated with tumor associated macrophages (TAMs) and highly invasive phenotypes. Moreover, HOXA7-siRNA inhibited migration and invasion *in vitro*, and downregulated MMP9 at the protein level in U251 glioma cells.

**METHODS:**

A cohort of 164 glioma specimens from the Chinese Glioma Genome Atlas (CGGA) array database were assessed as the training group. TCGA RNA-seq and GSE16011 mRNA array datasets were used for validation. Regression analyses and linear risk score assessment were performed for the identification of the three-gene signature comprising HOXA7, SLC2A4RG and MN1.

**CONCLUSIONS:**

We established a three-gene signature for lower grade gliomas, which could independently predict overall survival (OS) of lower grade glioma patients with higher sensitivity and specificity compared with other clinical characteristics. These findings indicate that the three-gene signature is a new prognostic model that could provide improved OS prediction and accurate therapies for lower grade glioma patients.

## INTRODUCTION

Gliomas are highly malignant intracranial tumors that account for more than half of primary central nerves system (CNS) cancers [1]. Despite the recent progress in neurosurgery, radiotherapy and chemotherapy, no great improvement of overall survival (OS) in gliomas patients has been reported in the past four decades [2].

Gliomas could be divided into four grades (I-IV) according to the World Health Organization (WHO) [3]. WHO grades II and III, including astrocytomas, oligodendrogliomas and mixed oligoastrocytomas are considered lower grade gliomas [4]. The common morphology-based classification could yield subjective differences regarding histological typing and grading between observers. In neuro-oncological practice, no clear national consensus for the diagnosis of adult gliomas has been reached [5], because of biological and clinical heterogeneity. Indeed, survival times in lower grade patients is totally different; some of them lived for more than 5 years in the CGGA array database, while others had really poor survival (less than 1 year).

With precision medicine becoming more popular, it would be helpful to lower grade gliomas patients to obtain a more accurate OS prediction and effective treatment [6]. With the development of high-throughput technologies, multiple biomarkers have been identified for predicting clinical results of cancer patients [7, 8]. In some malignant tumors, such as lung cancer and breast cancer, gene expression profiles of primary tumors have been established to predict OS [9, 10]. Many genes such as isocitrate dehydrogenase1 mutation (IDH1mut), epidermal growth factor receptor (EGFR) amplification, and Ki67expression have been included in gliomas to predict prognosis [4, 11, 12]. With deeper understanding of gliomas, it is acknowledged that polygenic abnormity, rather than single gene alterations, causes gliomas [13]. Here, we evaluated gene microarray expression profiles of three independent cohorts to establish a three-gene (HOXA7, SLC2A4RG and MN1) signature model. Using the new prognostic model, OS of lower grade gliomas patients could be predicted more objectively and accurately**.** Patients with different risk scores had various clinical and molecular features. The three-gene signature may have clinical implications in treating gliomas.

## RESULTS

### Discovery of the three-gene signature and prediction of survival in the CGGA database

**Figure 1:**
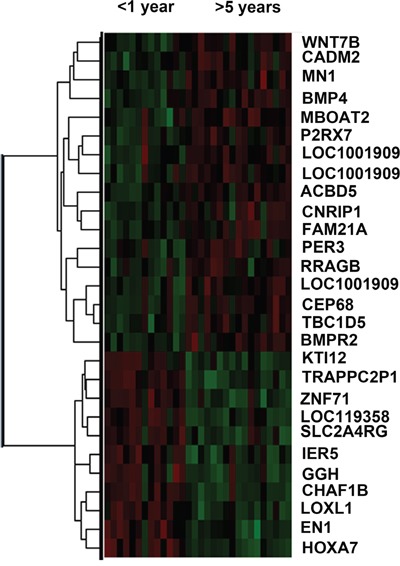
Heat map of differentially expressed genes in short (<1year) and long (>5year) survival groups Red, High expression; Green, Low expression.

**Figure 2:**
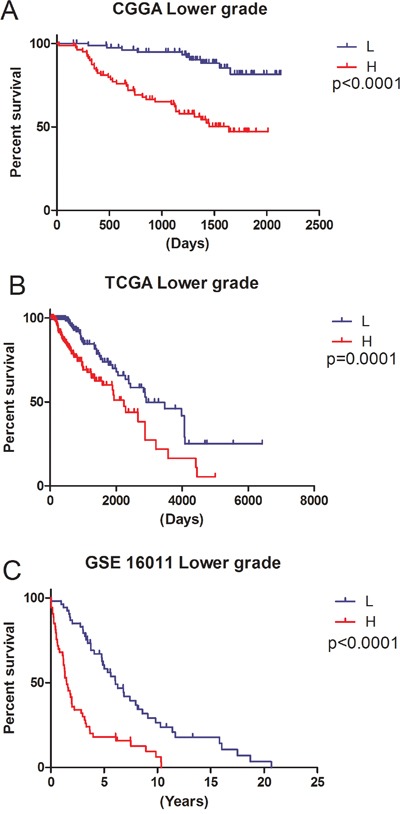
Ability of the three-gene signature to predict overall survival in the training and validation sets L, Low-risk group; H, High-risk group. **A.** CGGA mRNA array data (p<0.0001); **B.** TCGA RNA-seq data (p<0.0001); **C.** GSE16011 mRNA array data (p<0.0001).

In 164 CGGA lower grade gliomas samples, there was significant expression difference (*p*<0.05) in 28 genes (Figure 1 *p*<0.05) between short survival time (< 1 year) and long survival time (> 5 years). Then, Cox regression was carried out for analyzing genes in the training set (CGGA set); as a result, a total of 9 genes (CADM2, CEP68, CNRIP1, HOXA7, MBOAT2, MN1, SLC2A4RG, TBC1D5, TRAPPC2P1) were correlated with OS in grades II and III samples. For further assessment, univariate Cox regression was performed for the TCGA RNA-seq and GSE16011 mRNA array databases. Three genes (HOXA7, SLC2A4RG and MN1) were selected to establish a three-gene signature in lower grade gliomas (Table 1). Based on the three genes, we carried out the risk score assessment for predicting OS in lower grade gliomas [14]. In the training cohort, using the three-gene signature, we could divide lower grade glioma patients into low- and high-risk groups; a median risk score of −1.06 was chosen as cut off value for lower grade gliomas. Median survival times in the low- and high-risk groups were 1980 and 1610 days, respectively. High-risk group individuals showed significantly lower OS compared with low-risk group counterparts (*p*<0.05 Figure 2A); in addition, survival was markedly reduced in the high-risk group (Supplementary Table S1).

**Table 1:** Three genes associated significantly with overall survival (OS)

Gene	HR	95% CI	β	P
MN1	0.378	0.270-0.528	−0.974	<0.0001
HOXA7	1.379	1.260-1.509	0.321	<0.0001
SLC2A4RG	2.539	1.904-3.386	0.931	<0.0001

### Validation of the use fullness of the signature in two additional datasets

To verify the accuracy of the above results, TCGA RNA-seq and GSE 16011 array datasets were selected for assessment. The clinical characteristics of the two datasets are summarized in Supplementary Table S2. The same β value (Table 1) obtained from the CGGA mRNA array set was used to establish a risk score model. Based on the median risk score, we could divide validation sets of lower grade subjects in low- and high-risk groups. Concerning the signature's prognostic value, consistent with the training set, patients with high-risk scores had reduced survival time (Figure 2B and 2C).

### Clinical and molecular features of low and high risk lower grade glioma patients

To assess the relation of current risk groups with previous widely accepted factors and classification systems, basic clinical information and classical molecular markers were analyzed in the CGGA array database. The results are shown in Table 2; there were more patients (n=12) with Ki67 overexpression in the high-risk group compared with low-risk individuals (*p*=0.049). Patients with MMP9 overexpression were more likely found in high-risk group than those not overexpressing this protein (*p*=0.001). For grade, patients with anaplastic tumors were more likely found in the high-risk group (*p*<0.0001). Univariate and multivariate Cox regression analyses were further performed in the CGGA mRNA array set (Table 3), and the TCGA RNA-seq database was selected for validation (Table 4). In univariate regression, risk score (*p*<0.0001) along with grade (*p*<0.0001) and pre-operative KPS (*p*=0.041) could promote survival in lower grade gliomas. In multivariate regression, risk score was also significant (*p*=0.041) after adjusting for grade (*p*=0.147) and pre-operative KPS (*p*=0.119). For TCGA RNA-seq set, risk score was also significant (p<0.0001) in multivariate regression.

**Table 2:** Characteristics of patients in low risk and high-risk group in CGGA dataset

Clinical factors		Lower grade			P
		total	LR	HR	
		164	82	82	
Age	≤39	88	45	43	>0.05
	>39	76	37	39	
	NA	0			
Gender	F	71	33	38	>0.05
	M	93	49	44	
	NA	0			
Grade	G2	117	72	45	<0.0001
	G3	47	10	37	
	NA	0			
IDH1 mut	MUT	104	57	47	>0.05
	WT	56	23	33	
	NA	4			
TP53	MUT	24	8	16	>0.05
	WT	75	39	36	
	NA	65			
Ki67 exp	low	122	67	55	0.049
	high	17	5	12	
	NA	25			
MMP9	low	82	52	30	0.001
	high	82	30	52	
	NA	0			
PTEN mut	MUT	5	1	4	>0.05
	WT	90	44	46	
	NA	69			
1p/19q co-deletion	Yes	32	16	16	1
	No	132	66	66	
	NA	0			
EGFR amp	Yes	3	0	3	>0.05
	No	161	82	79	
	NA	0			
Pre-operative KPS	Yes	45	26	19	>0.05
	No	29	12	17	
	NA	90			

G2: grade 2; G3: grade 3; MT: mutational type; WT: wild type; IDHmut: isocitrate dehydrogenase mutation; TP53: a transcription factor protein which in humans is encoded by the TP53 gene. Ki67: a protein which in humans is encoded by the MKI67 gene; EGFRexp: epidermal growth factor receptor protein expression; MMP9: a migration and invasion marker encoded by the MMP9 protein; PTEN: phosphatase and tensin homolog deleted on chromosome 10. EGFR amp: epidermal growth factor receptor protein amplification; KPS: karnofsky performance score; HR: high-risk; LR: low-risk.

**Table 3:** Univariate and multivariate Cox analysis in CGGA lower grade glioma samples

Clinical factors	Univariate				Multivariate			
	HR	95%CL		P	HR	95%CL		P
		Lower	Upper			Lower	Upper	
Histology	0.564	0.316	1.006	0.05	0.581	0.169	1.997	0.388
Grade	4.309	2.419	7.676	<0.0001	2.262	0.751	6.81	0.147
Risk score	4.872	2.419	9.812	<0.0001	2.606	1.690	4.018	<0.0001
Preoperative KPS	0.319	0.107	0.952	0.041	0.700	0.200	2.451	0.577

Histology: astrocytoma vs oligodendroglioma and oligoastrocytoma; Grade: grade 2 vs grade 3; KPS: karnofsky performance score.

Risk score: high-risk vs low-risk.

**Table 4:** Univariate and multivariate Cox analysis in TCGA lower grade glioma samples

Clinical factors	Univatiate				Multivariate			
	HR	95%CI		P	HR	95%CI		P
		Lower	Upper			Lower	Upper	
Age	4.153	2.65	6.507	<0.0001	4.330	2.645	7.088	<0.0001
IDH1 mutation	0.344	0.226	0.524	<0.0001	0.591	0.376	0.929	0.023
Gender	0.98	0.654	1.468	0.921				
Grade	3.220	2.079	4.986	<0.0001	2.834	1.726	4.652	<0.0001
Histology	0.518	0.344	0.78	0.002	0.791	0.511	1.225	0.293
Risk score	2.21	1.46	3.344	<0.0001	3.384	2.085	5.492	<0.0001

Age: <41 vs ≥41; Gender: female vs male; Grade: grade 2 vs grade 3; Histology: astrocytoma vs oligodendroglioma and oligoastrocytoma.

Risk score: high-risk vs low-risk.

**Figure 3:**
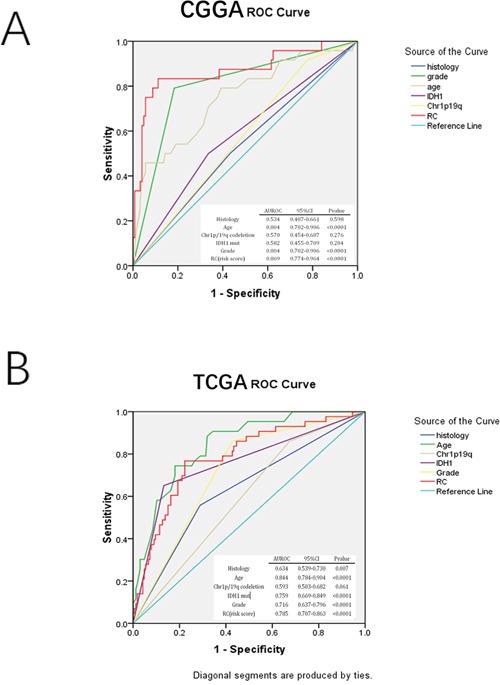
ROC analysis in assessing sensitivity and specificity for two-year survival prediction by the three-gene signature, histology, grade, age, IDH mut, and 1p/19q co-deletion in CGGA mRNA array and TCGA RNA-seq sets (largest validation set)

Receiver operating characteristic (ROC) assessment was performed to evaluate sensitivity and specificity of two year survival prediction for the three-gene signature, histology, grade, age, IDH1 mutation and 1p/19q co-deletion in the CGGA array dataset (training cohort) and TCGA RNA-seq dataset (the largest validation cohort). We found in the CGGA array dataset the area under the ROC curve (AUROC) for the3-gene set was the largest (0.869). In the TCGA RNA-seq dataset, AUROC value for the 3-gene set (0.785) was slightly smaller than that of age (0.844), but larger than that obtained with IDH1 mutation (0.759) and 1p/19q co-deletion (0.593). These results indicated that the three-gene signature might have a better predictive ability in predicting prognosis (Figure 3A–3B).

### Gene function interpretation and expression difference of the three genes in low- and high-risk groups

Little is known concerning the three genes (MN1, HOXA7, and SLC2A4RG) involved in the newly developed signature.

The MN1 gene with two CAG repeat sets is a tumor suppressor. A normal translocation in meningiomas could cause abnormal MN1expression. Meanwhile, MN1 inactivation could induce meningioma formation [15].

The HOXA7 gene belongs to cluster Aon chromosome 7 that controls morphogenesis, organogenesis and differentiation. As a transcription factor, HOXA7 regulates many critical genes involved in cancer cell proliferation and invasion [16]. For example, HOXA7 downregulates differentiation-specific genes during keratinocyte proliferation, a repression rescued by differentiation signals. HOXA7is highly similar to antennapedia (Antp) of *Drosophila* [17].

SLC2A4RG, a transcription factor activates the solute carrier family 2 member 4 gene; it also binds myocyte enhancer factor 2, another transcription factor, for upregulation of the latter [18].

There were marked differences in mRNA amounts between low- and high-risk individuals. Meanwhile, MN1 was overexpressed in the low risk group, with HOXA7 and SLC2A4RG overexpressed in the high risk group (Supplementary Figure S1).

### Functional annotation of the signature

**Figure 4:**
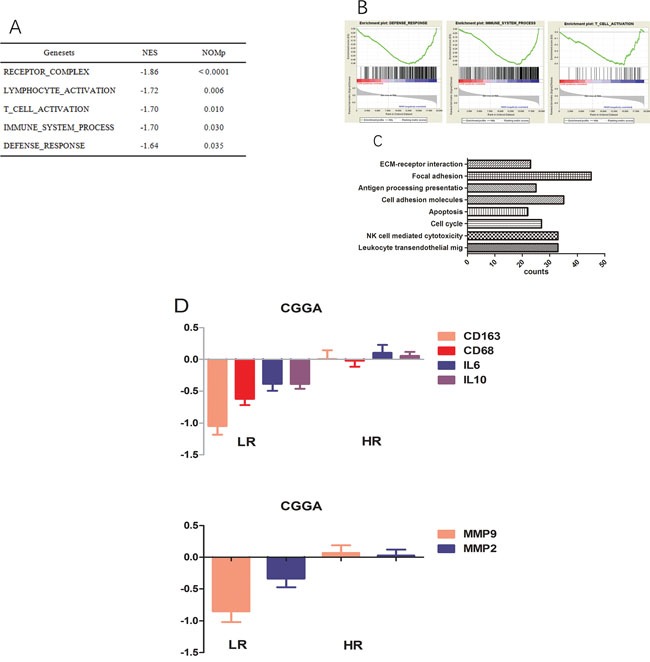
Functional annotation of risk groups **A.** Top five enriched pathways in the high-risk group, analysed by gene set enrichment analysis of CGGA data. **B.** Three representative plots of GSEA from A. **C.** GO analysis revealed significant associations of genes with increased expression in the high-risk group with eight main pathways. **D.** Genes encoding CD163, CD68, IL10, IL6, MMP2, and MMP9 are expressed at higher levels in the high-risk group.

We carried out GSEA and GO analyses to explain the significant OS difference observed between low and high risk score groups; as shown in Figure 4A–4C, T cell activation as well as receptor complex formation and the immune system process were highly enriched in the high risk group. For GO analysis, cell adhesion, focal adhesion, ECM-receptor interactions, leukocyte trans-endothelial migration, and natural killer cell dependent cytotoxicity were more pronounced in the high-risk group. Such findings may explain prognosis differences between both groups obtained using the signature.

### High risk score patients obtained by the three gene signature show tumor associated macrophages (TAMs)- and highly invasive phenotypes

The results of GSEA and GO analyses revealed that samples in the high-risk score group had TAMs activation and highly invasive phenotypes. in agreement, high-risk group patients showed higher levels of activated M2 macrophage related genes (CD68, CD163, IL10, and IL6, Figure 4D) compared with the low risk group in the training set. Likewise, well-known invasion markers (MMP2 and MMP9) were upregulated in the high-risk group (Figure 4D).

### HOXA7-siRNAinhibit migration and invasion *in vitro*

**Figure 5:**
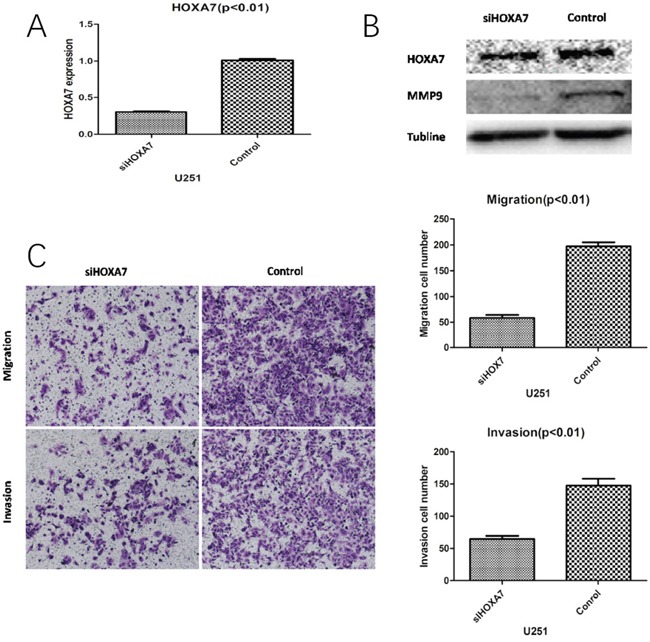
HOXA7 knockdown inhibits glioma cell migration and invasion *in vitro* **A.** RT-qPCR analysis showed that HOXA7-siRNA downregulated HOXA7 expression in U251 glioma cells. **B.** Western blot analysis showed the biological effects of HOXA7-siRNA. Downregulation of HOXA7 inhibited protein expression of MMP9, a well-known migration and invasion marker. **C.** HOXA7-siRNA inhibited U251 cell migration and invasion capacities (p<0.01). Representative images and accompanying statistical plots are shown.

Studies assessing HOXA7 in glioma are scared. Compared with control siRNA, HOXA7 mRNA and protein levels were considerably reduced in this study (Figure 5A and 5B). In addition, HOXA7-siRNA resulted in the downregulation of MMP9, a well-known migration and invasion marker (Figure 5B). Then, migration and Matrigel invasion assays were carried out using U251 cells. As shown in Figure 5C, HOXA7 knockdown via HOXA7-siRNAclearly inhibited glioma cell migration and invasion (Figure 5C).

## DISCUSSION

Gliomas, which are diagnosed based on histopathological criteria, are among the most frequent and aggressive cerebral tumors. However, the currently available histopathological classification has numerous shortcomings, because glioma is a polygenic and heterogeneous disease, suggesting that accurate molecular classification systems should be developed; this could help design more appropriate treatment plans for lower grade glioma cases. Considerable effort has been invested in glioma classification [19, 20], but little is known concerning lower grade gliomas. In this study, a three-gene signature (MN1, HOXA7, and SLC2A4RG) could divide lower grade glioma patients into low-and high-risk groups, with longer OS obtained in the former group.

An agreement has not been reached regarding the treatment of lower grade gliomas. Previous findings demonstrated that chemotherapy does not prolong OS in patients with isocitrate dehydrogenase 1-mutant (IDH1 mut) tumors in low grade gliomas [21]. Meanwhile, RTOG showed that combining PCV chemotherapy and radiotherapy improves median OS of patients with grade II gliomas who received no total tumor resection or were >40 years old [22]. The identified three-gene signature could provide an objective and accurate classification model. Furthermore, based on the three-gene signature, suitable therapies could be selected for different lower grade glioma patients. More aggressive therapeutic regimens could be used for high-risk patients, avoiding overtreatment of low-risk group patients.

In glioma mass, TAMs induced by glioma cells could provide a supportive microenvironment for tumor cell proliferation and invasion [23]. The high-risk group showed the TAMs phenotype, with higher levels of IL10, IL6, CD68 and CD163, classical markers for M2 TAMs [24]. Meanwhile, the expression levels of MMP9 and MMP2 were higher in high-risk patients compared with the low-risk group, suggesting gliomas had increased invasion potential in the former group. In the high-risk score group, there were more activated M2 TAMs than in low-risk patients, which may explain why gliomas in the high-risk group had increased invasion ability.

In summary, a three-gene signature was used to divide lower grade gliomas into low- and high-risk groups. High-risk group samples induced more M2 TAMs and had increased invasion potential. This signature constitutes a new prognostic model for classifying lower grade glioma patients more objectively and accurately compared with current methods, and could improve patient's quality of life.

## MATERIALS AND METHODS

### Datasets and patients

Samples were obtained from the Chinese Glioma Genome Atlas (CGGA) dataset (the training set);the TCGA RNA-seq database and GSE16011 array dataset were assessed as independent validation sets. Overall survival (OS) time (the period from surgery to death) was obtained through phone interviews with the patients and/or their relatives. Patients with survival shorter than 1year were considered short OS patients; those surviving for more than 5 years were classified in the long OS group.

### Real-time polymerase chain reaction (RT-PCR) and Western blot

RT-PCR and Western blot were carried out as recently described [25, 26]. RT-PCR primers were: HOXA7-F, 5′- TCGTATTATGTGAACGCGCTT-3′ and HOXA7-R, 5′-CAAGAAGTCGGCTCGGCATT −3′; GAPDH was used as a reference gene. Data were analyzed by the 2−ΔΔCt method. In Western blot experiments, TUBLINE was employed as a loading control.

### Cell culture and transfection of HOXA7-siRNA

Human glioma U251 cells were obtained from the American Type Culture Collection (ATCC), and maintained in DMEM containing 10% fetal bovine serum (FBS; Gibco, USA) and antibiotics (100 U/ml penicillin and 100 μg/mL streptomycin) in a humid environment with 5% CO2 at 37°C. HOXA7-siRNAs (sense, 5′-CAAACUACCUAUGUAUCCATT-3′; antisense, 5′-UGGAUACAUAGGUAGUUUGTT-3′) and a non-specific control siRNA (NC) were purchased from Genepharma (Shanghai, China), and transfected into U251 cells using Lipofectamine™ 2000 (Invitrogen, Shanghai, China). U251 cells were infected with HOXA7-siRNA for 48h before RT-PCR and Western blot assays [27].

### Gene set enrichment analysis (GSEA)

The Gene Set Enrichment Analysis software was used to perform GSEA. Based on median risk scores, the patients were divided into two groups: low- and high-risk groups. Then, ontology gene set from MSigDB was performed to assess enrichment gene sets for the three-gene signature.

### Statistical analyses

**Figure 6:**
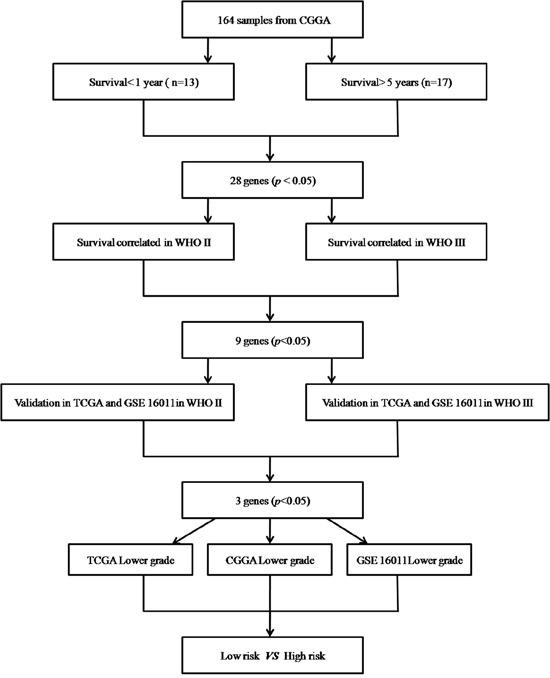
Procedure for selecting the 3 genes (HOXA7, SLC2A4RG and MN1) through whole genome mRNA profiling

Among the 164 lower grade glioma specimens, 117 grade II and 47 grade III samples were identified. These included 13 cases whose survival times were below 1 year and 17 who survived for more than 5 years. T-test and FDR correction were performed to compare differentially expressed genes between these two groups. FDR<0.05 was considered statistically significant. Univariate Cox regression analysis was carried out to assess these genes in predicting patient survival. A total of 1347 and 4224 genes showed significant differences (*p*< 0.05) in grade II and III patients, respectively. The differentially expressed genes common in both groups were validated by univariate Cox analysis in TCGA RNA-seq and GSE 16011 array databases. Three genes were adopted to establish the gene signature model (Figure 6). To assess the clinical value of the three-gene signature in predicting OS, a mathematical was generated. Specifically, as proposed previously [8], every patient was attributed a risk score based on linear combination of mRNA levels and Cox regression coefficients in lower grade glioma for the three genes:

Risk score = expr_gene1_ ×β_gene1 +_expr_gene2_ ×β_gene2 +_expr_gene3_ ×β_gene3_

Patients with no recorded survival time were excluded. For a gene with multiple probes, average expression was used to calculate the risk score. OS in high- and low-risk patient groups was estimated by the Kaplan-Meier method and 2-sided log-rank test in GraphPad Prism Version 6.01. Multiple Cox regression analysis was used to assess independent contributions to OS prediction after adjusting for other classical clinicopathologic variables [28].

## SUPPLEMENTARY MATERIALS FIGURE AND TABLES


