# Supplementary material for: Classifying lower grade glioma cases according to whole genome gene expression
Source: Oncotarget. 2016 Sep 22;7(45):74031–42. doi: 10.18632/oncotarget.12188 (PMC5342033; doi:10.18632/oncotarget.12188)
Supplement: Supplementary file 1 [file oncotarget-07-74031-s001.pdf]

Classifying lower grade glioma cases according to whole genome gene expression

SUPPLEMENTARY FIGURE AND TABLES

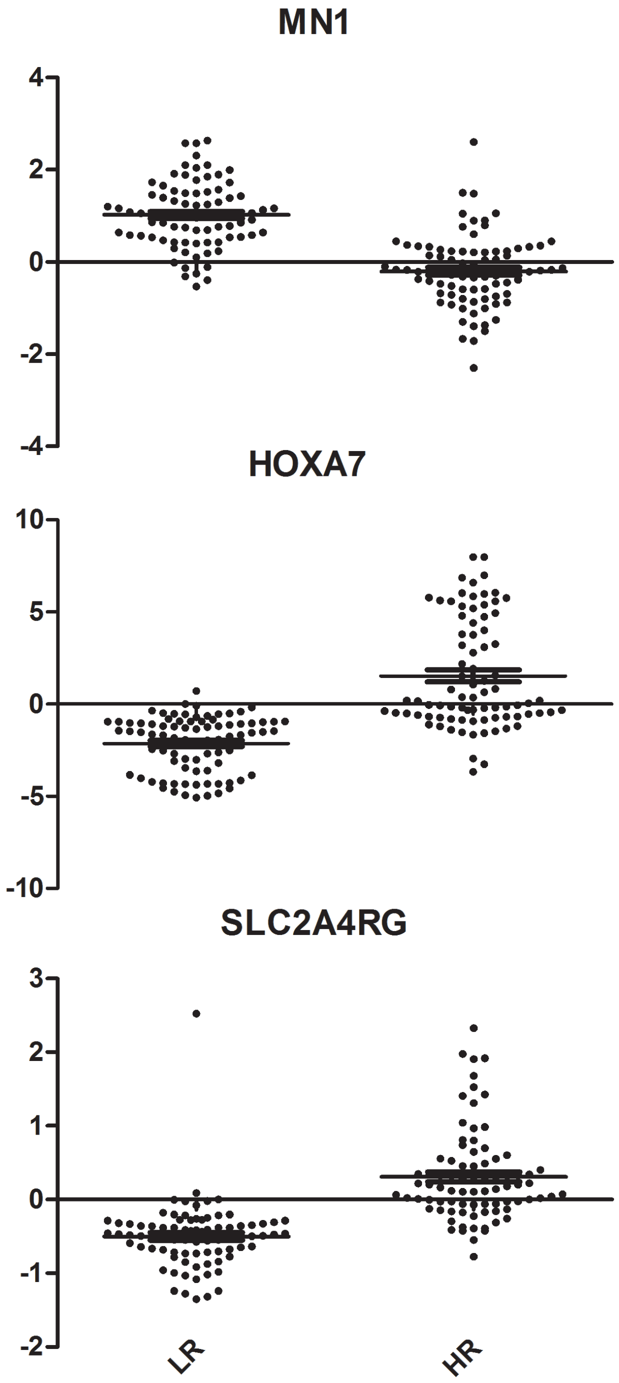

Supplementary Figure S1: Expression differences of the three-gene signature in CGGA data.

Supplementary Table S1: Survival rate of patients in LR and HR

		LR%	HR%
**OS**	Median (days)	1980	1610
	6 months	99	85
	12 months	97	77
	24 months	95	67
	36 months	92	58

OS: overall survival; LR: low risk; HR: high risk.

Supplementary Table S2: Clinicopathological characteristics of patients with lower grade glioma in the validation datasets (TCGA RNA-seq and GSE 16011)

**Sample size**	**TCGA**				**GSE16011**			
		**Total number**	**Group**			**Total number**	**Group**	
		466	LR	HR		133	LR	HR
	Female	211	97	114	Female	44	24	20
Gender	Male	255	136	119	Male	89	42	47
	NA	0			NA	0		
	G2	232	103	129	G2	48	26	22
Grade	G3	234	130	104	G3	85	40	45
	NA	0			NA	0		
	<41	231	119	112	<43	62	31	31
Age	≥41	234	113	121	≥43	71	35	36
	NA	1			NA	0		
	A	170	55	115	A	42	14	28
Histology	O	121	61	60	O	60	41	19
	OA	175	117	58	OA	31	11	20
	NA	0			NA	0		
	≤A7	85	32	53	≤37	58	27	31
KPS	>80	206	110	96	>80	75	39	36
	NA	165			NA	0		

G2: grade 2; G3: grade 3; A: astrocytoma and anaplastic astrocytoma ; O: oligodendroglioma and anaplastic oligodendroglioma; OA: oligoastrocytoma and anaplastic oligoastrocytoma; KPS: karnofsky performance score; HR: high-risk; LR: low-risk.
